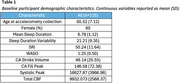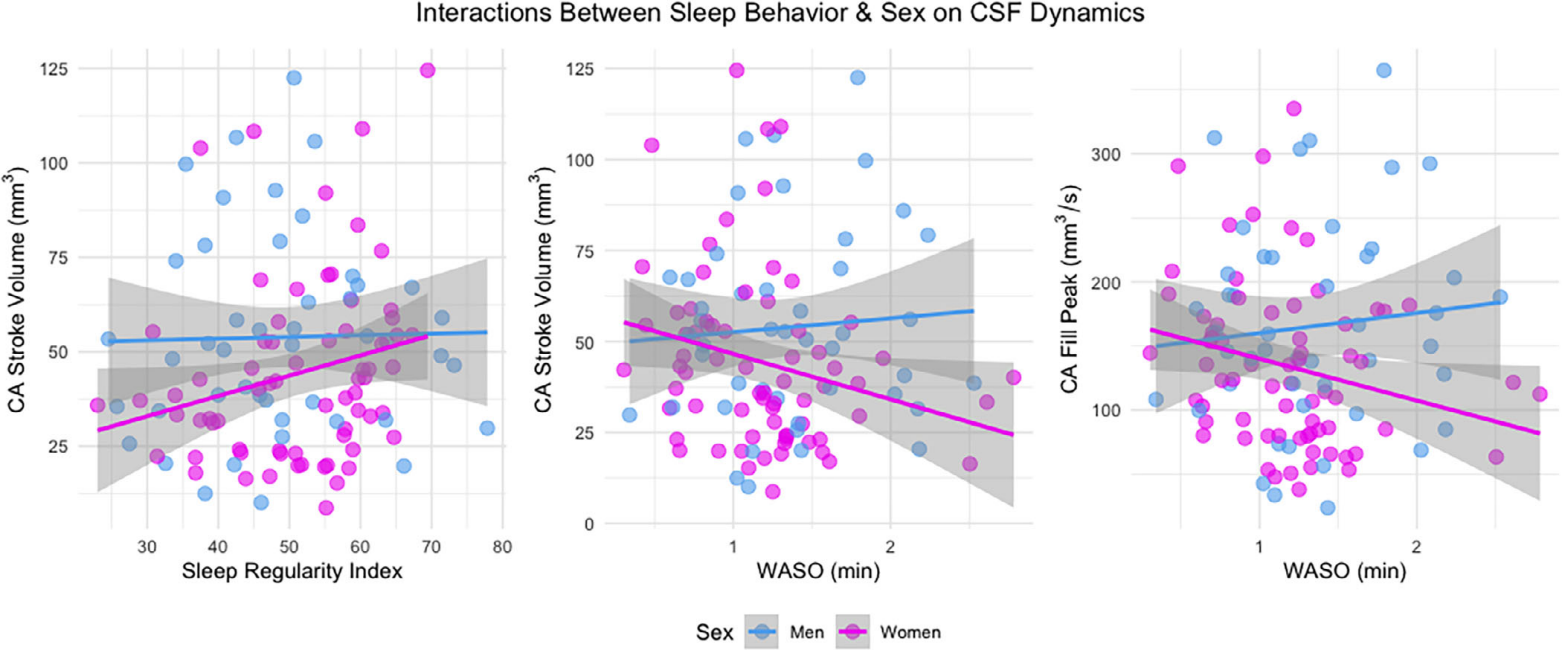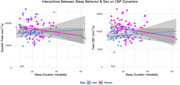# Sex‐specific effects of objective sleep behavior on cerebrospinal fluid and cerebral blood flow dynamics in older adults

**DOI:** 10.1002/alz70856_106096

**Published:** 2026-01-08

**Authors:** Yilei Dong, Laura E. Fenton, Ashwin Sakhare, Joy Stradford, Teresa Monreal, A. Lisette Isenberg, Judy Pa

**Affiliations:** ^1^ Alzheimer's Disease Cooperative Study (ADCS), University of California, San Diego, La Jolla, CA, USA; ^2^ University of Southern California, Los Angeles, CA, USA; ^3^ SDSU/UCSD Joint Doctoral Program in Clinical Psychology, University of California, San Diego, La Jolla, CA, USA; ^4^ Mark and Mary Stevens Neuroimaging and Informatics Institute, Keck School of Medicine, University of Southern California, Marina del Rey, CA, USA; ^5^ Alzheimer's Disease Cooperative Study (ADCS), University of California, San Diego, La Jolla, CA, USA; ^6^ Neuroscience Graduate Program, University of California San Diego, La Jolla, CA, USA

## Abstract

**Background:**

The glymphatic system clears metabolic waste (e.g., beta‐amyloid) via cerebrospinal fluid (CSF) and cerebral blood flow (CBF) and has been shown to increase during sleep. This activity may help reduce dementia risk. However, associations between sleep behavior and CSF/CBF flow dynamics remain unclear. This study examined these associations and further explored sex‐by‐sleep interactions on flow dynamics.

**Method:**

Participants included 115 healthy older adults (mean age: 65.52±7.12 years, 60% female). Sleep behavior was objectively measured with a GENEActiv accelerometer over an average of 27.73±6.52 days. Sleep variables included mean sleep duration, sleep duration variability (SDV), sleep regularity index (SRI), and wake after sleep onset (WASO). CSF and CBF flow measurements were assessed via 2D cine PC‐MRI pulse sequencing with retrospective cardiac gating at the cerebral aqueduct (CA) and C2‐C3 arteries. Flow variables included CA stroke volume, CA fill peak, systolic peak, and total CBF. Partial correlation models, adjusted for sex and age, examined associations between sleep and flow dynamics. Multivariable regression models tested sex‐by‐sleep interactions, followed by sex‐stratified analyses for interactions at *p* ≤ 0.20.

**Result:**

Among all participants, no significant correlations were observed between sleep behavior and flow dynamics. However, there were several notable sex‐by‐sleep interactions. There was an interaction between SRI and sex on CA stroke volume (β=.56, *p* = .15), where higher SRI correlated with greater CA stroke volume in women (r=.24, *p* = .05). There was an interaction between WASO and sex on CA stroke volume (β=‐14.99, *p* = .10) and CA fill peak (β=‐45.55, *p* = .09), where greater WASO was trend‐level associated with lower CA stroke volume (*r* =‐.24, *p* = .05) and CA fill peak (r=‐.24, *p* = .05) in women. There was also an interaction between SDV and sex on systolic peak (β=‐105.76, *p* = .19) and total CBF (β=‐81.95, *p* = .10). Results of sex‐stratified analyses revealed that, in women, greater SDV was significantly associated with lower total CBF (r=‐.24, *p* = .05) and trending towards an association with lower systolic peak (r=‐.21, *p* = .10). No significant relationships were observed in men.

**Conclusion:**

These findings suggest better sleep behavior, particularly in women, is linked to better CSF and CBF flow dynamics.